# D-PAttNet: Dynamic Patch-Attentive Deep Network for Action Unit Detection

**DOI:** 10.3389/fcomp.2019.00011

**Published:** 2019-11-29

**Authors:** Itir Onal Ertugrul, Le Yang, László A. Jeni, Jeffrey F. Cohn

**Affiliations:** 1 Robotics Institute, Carnegie Mellon University, Pittsburgh, PA, United States,; 2 School of Computer Science, Northwestern Polytechnical University, Xian, China,; 3 Department of Psychology, University of Pittsburgh, Pittsburgh, PA, United States

**Keywords:** action unit detection, 3D face registration, 3D-CNN, sigmoidal attention, patch-based

## Abstract

Facial action units (AUs) relate to specific local facial regions. Recent efforts in automated AU detection have focused on learning the facial patch representations to detect specific AUs. These efforts have encountered three hurdles. First, they implicitly assume that facial patches are robust to head rotation; yet non-frontal rotation is common. Second, mappings between AUs and patches are defined a priori, which ignores co-occurrences among AUs. And third, the dynamics of AUs are either ignored or modeled sequentially rather than simultaneously as in human perception. Inspired by recent advances in human perception, we propose a dynamic patch-attentive deep network, called D-PAttNet, for AU detection that (i) controls for 3D head and face rotation, (ii) learns mappings of patches to AUs, and (iii) models spatiotemporal dynamics. D-PAttNet approach significantly improves upon existing state of the art.

## 1. INTRODUCTION

Facial actions communicate intention, emotion, and physical state (Tian et al., 2001). The most comprehensive method to annotate facial action is the anatomically-based Facial Action Coding System (FACS) (Ekman et al., 2002). Action units defined in FACS correspond to facial muscle movements that individually or in combination can describe nearly all possible facial expressions. Automated detection of AUs has become a crucial computer vision problem.

The core of the human neural system for face and facial action perception consists of three bilateral regions, the occipital face area (OFA), fusiform face area (FFA), and superior temporal sulcus (STS) (Haxby et al., 2000). Previous work suggests that the OFA represents face parts, including eyes, nose, and mouth, in the early stage of face perception (Liu et al., 2010; Nichols et al., 2010; Arcurio et al., 2012). At a higher-level, the FFA performs holistic processing and representations of identity (George et al., 1999; Hoffman and Haxby, 2000). The STS is sensitive to facial dynamics and involves the representation of changeable aspects of faces such as expression, lip movement, and eye gaze (Hoffman and Haxby, 2000). The anatomical location of OFA suggests that it provides input to both the FFA and STS. This system is consistent with hierarchical models (Grill-Spector and Malach, 2004; Fairhall and Ishai, 2006) that propose that complex visual objects are recognized via a series of stages in which features of increasing complexity are extracted and analyzed at progressively higher levels of the visual processing stream (Pitcher et al., 2011). The success of many human-inspired approaches in machine learning urges the following question: Can we model machine perception of facial actions with a hierarchical system analogous to the suggested models of human perception of faces and facial action?

Recent approaches to facial action detection have begun to address this question. Analogous to the OFA in human face perception, region learning, or what is referred to as patch learning, separately processes specific facial regions. This work is informed by the observation that the human face is more structured than many other natural images and different face regions have different local statistics (Zhao et al., 2016b). Variation in local statistics stems from both structural features and transient facial muscle contraction and relaxation. Facial action units (AUs), which are anatomically based, are responsible for muscle contraction and relaxation. For instance, tightening of the eye aperture results from contraction of the inner portion of the orbicularis oculi muscle, which is AU7. Performing AU7 will change the appearance of eye corners and not mouth regions. When the goal is to detect AU7, it is natural to look around eye region more than mouth region. Therefore, due to the locality of AUs, some facial regions are more important than others to detect specific AUs (Zhao et al., 2016a). Thus, patch learning approaches have components for representing facial parts. These local parts then are integrated holistically in mechanisms analogous to the FFA in human face perception.

Patches have been defined in one of two principal ways. One is with respect to fixed grids (Liu et al., 2014). The other is centered around facial landmarks (Zhao et al., 2016a). Both approaches assume that patches are invariant to head rotation. That is, when the head moves or rotates, patches are assumed to maintain consistent semantic correspondence. This assumption often is violated. Faces look very different from different poses. Because most registration techniques treat the face as a 2D object, they are unable to accommodate 3D head rotation. In this work, we address this problem.

Another problem is that mappings between AUs and patches are defined a priori, and the mappings often fail to exploit co-occurrences among AUs. We know that some AUs frequently co-occur, while others inhibit the activity of others. AU6 (cheek raiser) and AU12 (oblique lip-corner puller) occur together in both Duchenne smiles and in pain expressions. AU24, which presses the lips together, inhibits dropping of the jaw (AU27). Because appearance changes in different facial regions are likely to contribute to the prediction of co-occurring AUs, it may be advantageous to weight the significance of patches to detection of specific AUs. Some patch-based AU detection methods fail to weight the contribution of each patch (Zhao et al., 2016b). A few of them do by using either regularization on the shallow representation of patches (Zhao et al., 2016a) or pre-defined attention masks in CNN (Jaiswal and Valstar, 2016; Sanchez et al., 2018), which often ignore AU correlations. Below, we show that AU detection can be improved by learning attention maps empirically to accommodate AU correlations.

The STS is sensitive to dynamic change in facial parts, and a number of studies have reported that dynamic information contributes to expression perception (Ambadar et al., 2005; Bould et al., 2008; Kätsyri and Sams, 2008; Horstmann and Ansorge, 2009). Yet, most recent work in machine perception of AUs ignores motion information or dynamics. In static approaches, each video frame is considered independently and outside of its temporal context. Temporal context may matter little for strong AUs but for subtle AUs lack of dynamics weakens the detection. Human observers have difficulty perceiving subtle AUs when motion information is missing (Ambadar et al., 2005). The same may be true for automated AU detection. When dynamics has been considered, spatial and temporal information typically is handled sequentially. For instance, a CNN represents spatial information and then LSTM models temporal information (Jaiswal and Valstar, 2016; Chu et al., 2017; Li et al., 2017). In human perception, on the other hand, spatiotemporal information may be processed tightly integrated.

Informed by human face perception and facial anatomy and dynamics, we propose a dynamic patch-attentive deep network (D-PAttNet) for AU detection. D-PAttNet jointly learns static and dynamic patch representations and weights them for AU detection. We first apply 3D registration to reduce changes from head movement and preserve facial actions that would be distorted by change in pose. Then, we crop local patches that contain the same facial parts across frames and that are informative for detection of specific AUs. We encode patches with individual 2D and 3D CNNs and obtain local representations that capture spatiotemporal information. Inspired by the recent success of attention mechanisms in various tasks including neural machine translation (Luong et al., 2015), text classification (Yang et al., 2016), and object detection (Rodríguez et al., 2018), we then introduce an attention mechanism to weight the importance of patches in detecting specific AUs. Since our network is trained in an end-to-end manner, the network itself learns (i) static and dynamic encoding of patches and (ii) the degree of attention to those patches to maximize AU detection. Unlike state-of-the-art attention approaches, which employ softmax activation function to “select” where to attend, we propose sigmoidal attention to allow networks to attend to multiple patches when needed.

The contributions of this paper are:

- An end-to-end trainable dynamic patch-attentive deep network that learns to encode static and dynamic patch information and learns to attend to specific patches for the detection of specific AUs.
- A sigmoidal attention mechanism that allows multiple static and dynamic patch encodings to contribute to the prediction of specific AUs.
- Relative to state of the art, an increase of 2.1% performance in F1-score and 0.7% performance in AUC.

## 2. RELATED WORK

### 2.1. Using Dynamics for AU Detection

Most AU detection approaches model frames individually and ignore the temporal dependencies among them (Chu et al., 2013; Zeng et al., 2015; Zhao et al., 2018; Onal Ertugrul et al., 2019a,c). Valstar and Pantic (2007) combine Support Vector Machines and Hidden Markov Models to incorporate temporal information. Gonzalez et al. (2015) propose a hidden semi-Markov model (HSMM) and variable duration semi-Markov model (VDHMM) to recognize AU dynamics. Koelstra et al. (2010) present a dynamic texture based approach that combines a discriminative, frame-based GentleBoost classifier with a dynamic, generative HMM model for temporal AU classification. Yang et al. (2009) extract temporal information of facial expressions using dynamic haar-like features and uses AdaBoost to select highly discriminating subset of these for AU recognition. Jeni et al. (2014) represent the spatio-temporal organization of expressions with time-series of shape and appearance descriptors and uses time-warping methods to classify different facial actions.

Recently, deep approaches have been proposed to model temporal information for AU detection. Chu et al. (2017) propose an architecture that combines convolutional neural network (CNN) and long short-term memory network (LSTM) for multilabel AU detection. In this architecture, CNN is used to learn spatial representations within frames while LSTM is used to model temporal dynamics among frames. Similarly, Jaiswal and Valstar (2016) use CNN to obtain spatial representations of facial parts cropped from the whole face using binary masks and used Bi-directional LSTM to learn the dynamics of facial parts for AU detection. Li et al. (2017) propose an adaptive region cropping based multi-label learning with deep recurrent net, which is based on combining region-based CNN (RCNN) with LSTM. Although a few deep approaches considering dynamics for AU detection have been proposed, many efforts have been devoted to incorporate dynamics in deep models for emotion recognition (Fan et al., 2016; Vielzeuf et al., 2017; Kollias and Zafeiriou, 2018; Liu et al., 2018; Lu et al., 2018). However, focusing on detecting action units is crucial since FACS is a comprehensive, anatomically-based system which describes all visually discernible facial movement and provides an objective measure.

As noted above, both shallow and deep AU detection approaches (e.g., SVM and 2D CNN) alike combine spatial and temporal information sequentially. Temporal representation is added only after spatial representation. In contrast, in human perception spatiotemporal processing is tightly integrated.

In a recent study, Yang et al. (2019) have proposed to model spatiotemporal information combining 2D-CNN with 3D-CNN for frame-level AU detection. However, whole video sequences are fed as input to 3D-CNN part to provide summary information about the entire video while modeling each frame. They do not consider modeling the local dynamics of segments, which is more informative to detect AUs.

### 2.2. Patch Learning

Traditional AU detection methods are based on (i) extracting appearance (Jiang et al., 2011; Eleftheriadis et al., 2015; Baltrusaitis et al., 2018) or geometric features (Lucey et al., 2007; Du et al., 2014) from the whole face and (ii) obtaining shallow representations as histograms of these features, thus ignoring the specificity of facial parts to AUs (Shojaeilangari et al., 2015). Deep approaches using whole face to train CNNs (Hammal et al., 2017; Onal Ertugrul et al., 2019a) also ignore the specificity of facial parts. More recent approaches focus on obtaining local representations using *patch learning*. Some of these approaches divide the face image into uniform grids (Liu et al., 2014; Zhong et al., 2015; Zhao et al., 2016b) while others define patches around facial parts (Corneanu et al., 2018) or facial landmarks (Zhao et al., 2016a). Among them, Liu et al. (2014) divide a face image into non-overlapping patches and categorize them into common and specific patches to describe different expressions. Zhong et al. (2015) identify active patches common to multiple expressions and specific to an individual expression using a multi-task sparse learning framework. Zhao et al. (2016b) use a regional connected convolutional layer that learns specific convolutional filters from sub-areas of the input. Corneanu et al. (2018) crop patches containing facial parts, train separate classifiers for each part and fuse the decisions of classifiers using structured learning. Zhao et al. (2016a) describe overlapping patches centered at facial landmarks, obtain shallow representations of patches and identify informative patches using a multi-label learning framework. These studies generally pre-process their frames to remove roll rotation. None of the aforementioned studies perform a 3D face registration to remove pitch and yaw rotation. Hence, patches cropped from different frames are likely to contain variable facial regions under pose. Only in a recent study, Onal Ertugrul et al. (2019b) cropped patches from 3D-registered faces for AU detection from static frames.

### 2.3. Regional Attention

As described in FACS (Ekman et al., 2002), AUs relate to specific regions of human faces. Motivated by this fact, recent studies aim to highlight information obtained from specific facial regions to detect specific AUs. Zhao et al. (2016a) employ patch regularization to eliminate the effect of non-informative shallow patch representations. Taheri et al. (2014) learn a dictionary per AU using local features extracted from predefined AU semantic regions on faces performing that AU. Jaiswal and Valstar (2016) use a pre-defined binary mask created to select a relevant region for a particular AU and pass it to a convolutional and bidirectional Long Short-Term Memory (LSTM) neural network. Li et al. (2018) design an attention map using the facial key points and AU centers to enforce their CNN-based architecture to focus more on these AU centers. Sanchez et al. (2018) generate heatmaps for a target AU, by estimating the facial landmarks and drawing a 2D Gaussian around the points where the AU is known to cause changes. They train Hourglass network to estimate AU intensity. Shao et al. (2018) employ an initial attention map, created based on AU centers and refine it to jointly perform AU detection and face alignment. These studies have mechanisms to enforce their models to focus on pre-defined regions. They do not have a learned attention mechanism, in which the network decides where to attend itself for each AU. In a recent work, Onal Ertugrul et al. (2019b) has proposed a mechanism which learns to attend to significant patches from their static encodings.

## 3. METHODS

**FIGURE 1 |:**
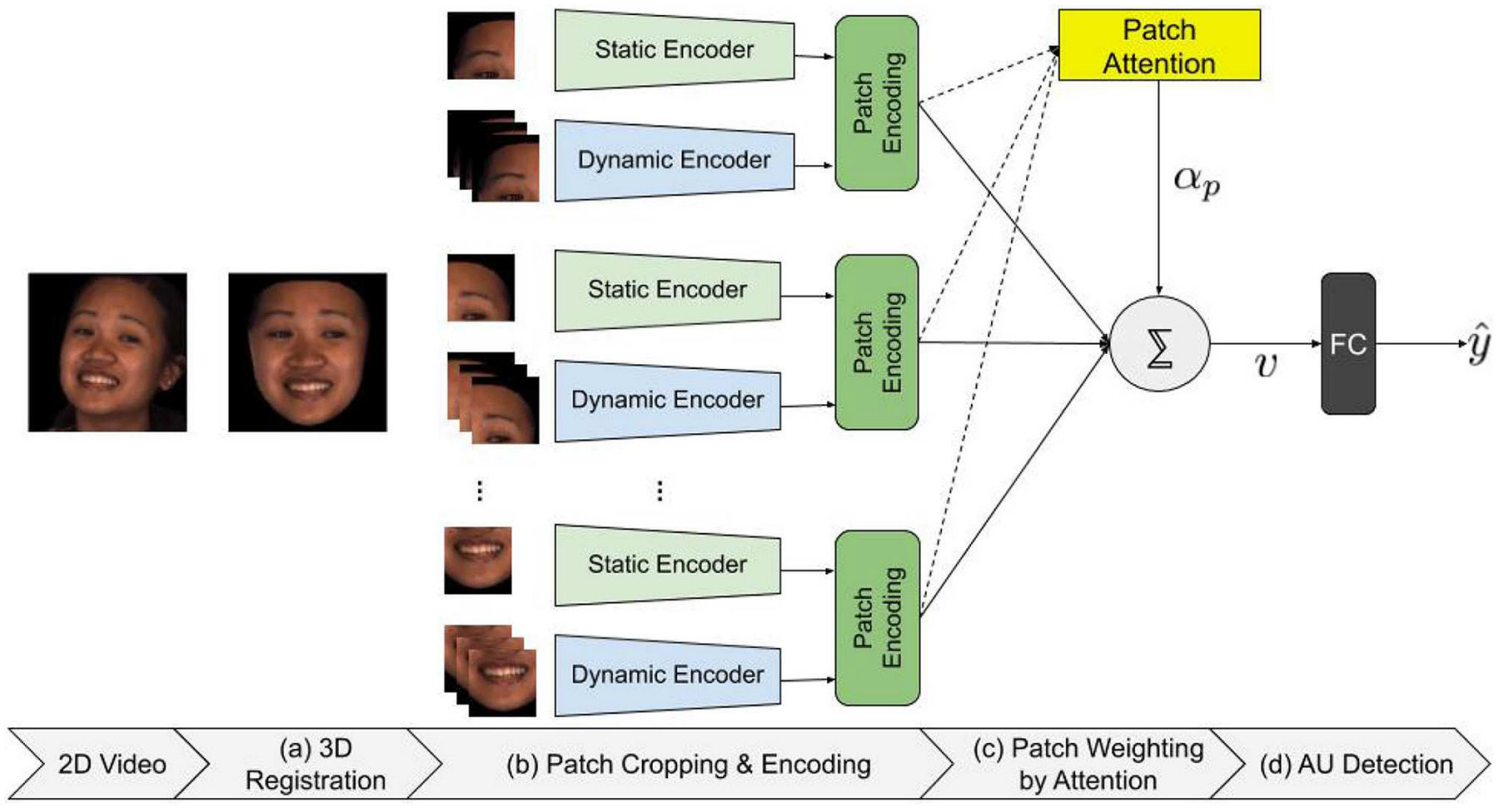
Proposed D-PAttNet approach. **(a)** A dense set of facial landmarks is estimated and a dense 3D mesh of the face is reconstructed. **(b)** Patches containing facial regions related to specific AUs are cropped and fed to different CNNs for encoding. For each patch, 2D-CNN is used to encode static frame-level information and 3D-CNN is used to encode dynamic, segment-level information. Patch encoding is obtained by concatenating static and dynamic encoding. **(c)** Patches are weighted by sigmoidal attention mechanism to detect specific AUs. **(d)** Face encodings are fed to a fully connected layer (FC) to detect AUs.

Figure 1 shows the components of the proposed dynamic patch-attentive network (D-PAttNet) architecture. First, we perform dense 3D registration from 2D videos (Figure 1a). Then, we crop patches containing local facial parts. For each patch location, we use a separate 2D-CNN to encode local, static information and 3D-CNN to encode local, dynamic information. We concatenate static and dynamic encoding to obtain patch encoding (Figure 1b). We employ a sigmoidal attention mechanism to weight the contribution of each patch to detect specific AUs (Figure 1c). Finally, using the final face encoding, we detect 12 AUs (Figure 1d). In the following, we describe in detail, the different components of the proposed D-PAttNet approach.

### 3.1. 3D Face Registration

We track and normalize videos using ZFace (Jeni et al., 2015, 2017), a real-time face alignment software that accomplishes dense 3D registration from 2D videos and images without requiring person-specific training. ZFace performs a canonical 3D normalization that minimizes appearance changes from head movement and maximizes changes from expressions. First, it uses dense cascade-regression-based face alignment to estimate a dense set of 1,024 facial landmarks. Then a part-based 3D deformable model is applied to reconstruct a dense 3D mesh of the face. Face images are normalized in terms of pitch, yaw and roll rotation and scale and then centered. At the output of this step, video resolution is 512 × 512 with an interocular distance (IOD) of about 100 pixels.

### 3.2. Patch Cropping and Encoding

The 3D face registration step ensures that faces in all frames of all individuals are registered to the same template and that same landmarks (facial parts) in all frames are very close to each other. This step allows us to identify the locations of face parts and crop patches containing the same face parts for all frames.

**FIGURE 2 |:**
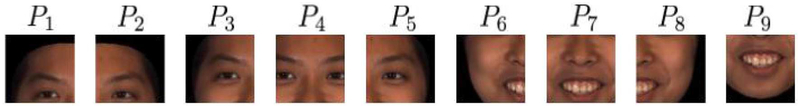
Cropped patches from 3D registered face images.

Patch locations are identified using the domain knowledge of human FACS coders and based on the FACS manual (Ekman et al., 2002). We identify *N* = 9 patches given in Figure 2 with the aim to cover specific face parts that are deformed during the appearance of specific AUs, namely right eyebrow (*P*_1_), left eyebrow (*P*_2_), right eye (*P*_3_), region between eyebrows and nose root (*P*_4_), left eye (*P*_5_), right cheek and lip corner (*P*_6_), nose and upper mouth (*P*_7_), left cheek and lip corner (*P*_8_), and mouth and chin (*P*_9_). Then, we crop *N* = 9 patches using the same identified locations from all frames in the dataset. The size of each RGB patch is 100 × 100 pixels.

#### 3.2.1. Static Patch Encoding

We use 2D-CNNs to encode static information. Input to each 2D-CNN is a single patch. We feed patches cropped from each of the nine locations to a different static encoder so that each encoder aims to learn representations of local face parts. Each of the nine static encoders has an identical architecture, which includes three convolutional layers and 1 fully connected layer. At the output of static encoders, we obtain *M*-dimensional vector representations of local patches.

#### 3.2.2. Dynamic Patch Encoding

We use 3D-CNNs to encode dynamic information. We feed a patch sequence of length *T* as input to each 3D-CNN. Note that, each patch sequence contains the current patch fed to 2D-CNN and *T* − 1 patches preceding the current patch. Similar to static encoders, we feed patch sequences cropped from each of the nine locations to a different dynamic encoder so that each encoder aims to learn dynamic representations of local face parts. 3D-CNNs have the same architectures as 2D-CNNs except 2D convolution layers are replaced by 3D convolution layers. At the output of dynamic encoders, we obtain *M*-dimensional vector representations of local patches.

After we obtain static and dynamic encoding of patches, we concatenate them and have a 2M-dimensional patch encoding.

### 3.3. Patch Weighting by Sigmoidal Attention Mechanism

Different face patches contribute unequally to the face representation to predict AUs. In order to weight the contribution of patch encodings, we use an attention mechanism. An attention mechanism aggregates the representation of the informative patch encodings to form a face encoding. Let *e*_*p*_ be the encoding of patch *p* obtained by concatenating the outputs of 2D and 3D CNNs. First, patch encoding *e*_*p*_ is fed to a one-layer MLP to obtain hidden representation *h*_*p*_ of *e*_*p*_ as follows:

(1) $$h_{p}=\text{tanh}\left(W_{f}e_{p}+b_{f}\right)$$

where *W*_*f*_ and *b*_*f*_ are the weight and bias parameters of the MLP, respectively. Then, the importance of each patch is measured by the similarity between *h*_*p*_ and a patch level context vector *c*_*f*_. In order to normalize the importance of patches to the range [0,1] and obtain attention weight *α*_*p*_, we apply sigmoid function as follows:

(2) $$\alpha_{p}=\frac{1}{1+\text{exp}\left(-h_{p}^{T}c_{f}\right)}$$

If a patch representation is similar to context vector, their inner product will give a large value, and sigmoid output will be closer to 1. On the other hand, if a patch representation is very different from context vector, then their inner product will be close to zero, and the sigmoid output will also be close to zero (meaning that given patch is not important to detect the AU). Therefore, patch level context vector *c*_*f*_ can be interpreted as the high level representation of fixed query “What are the informative patches to predict a specific AU?” It is randomly initialized and learned during training. Finally, we obtain face encoding *v* as a weighted sum of patch encodings *e*_*p*_ as:

(3) $$v=\underset{p}{\sum }\alpha_{p}e_{p}$$

Note that, it is typical to use softmax activation function for normalization in attention mechanisms employed in many NLP tasks. One such task is neural machine translation, where the network is trained to attend to one word (or a few words, but not to the others) to obtain the corresponding translation of the word. Output of softmax function can be used to represent a categorical distribution. In our case, we aim to allow multiple patches to contribute to predict a specific AU. Therefore, instead of softmax, we used sigmoid activation function which allows for multiple selection with a collection of Bernoulli random variables.

### 3.4. AU Detection

Face encoding *v* is a high level representation of the face that is used for AU detection. To *v* we apply ReLU for non-linearity and have a fully connected layer to predict the occurrence of AUs. We train individual networks for each AU. We apply sigmoid function and use weighted binary cross-entropy loss as follows:

(4) $$L=-\text{ylog}\left(\hat{y}\right)w_{\text{pos}}-\left(1-y\right)\text{log}\left(1-\hat{y}\right)$$

where *y* denotes actual AU occurrence, ŷ denotes predicted AU occurrence. *w*_*pos*_ is the weight that is used for adjusting positive error relative to negative error.

## 4. EXPERIMENTS

### 4.1. Dataset

**FIGURE 3 |:**
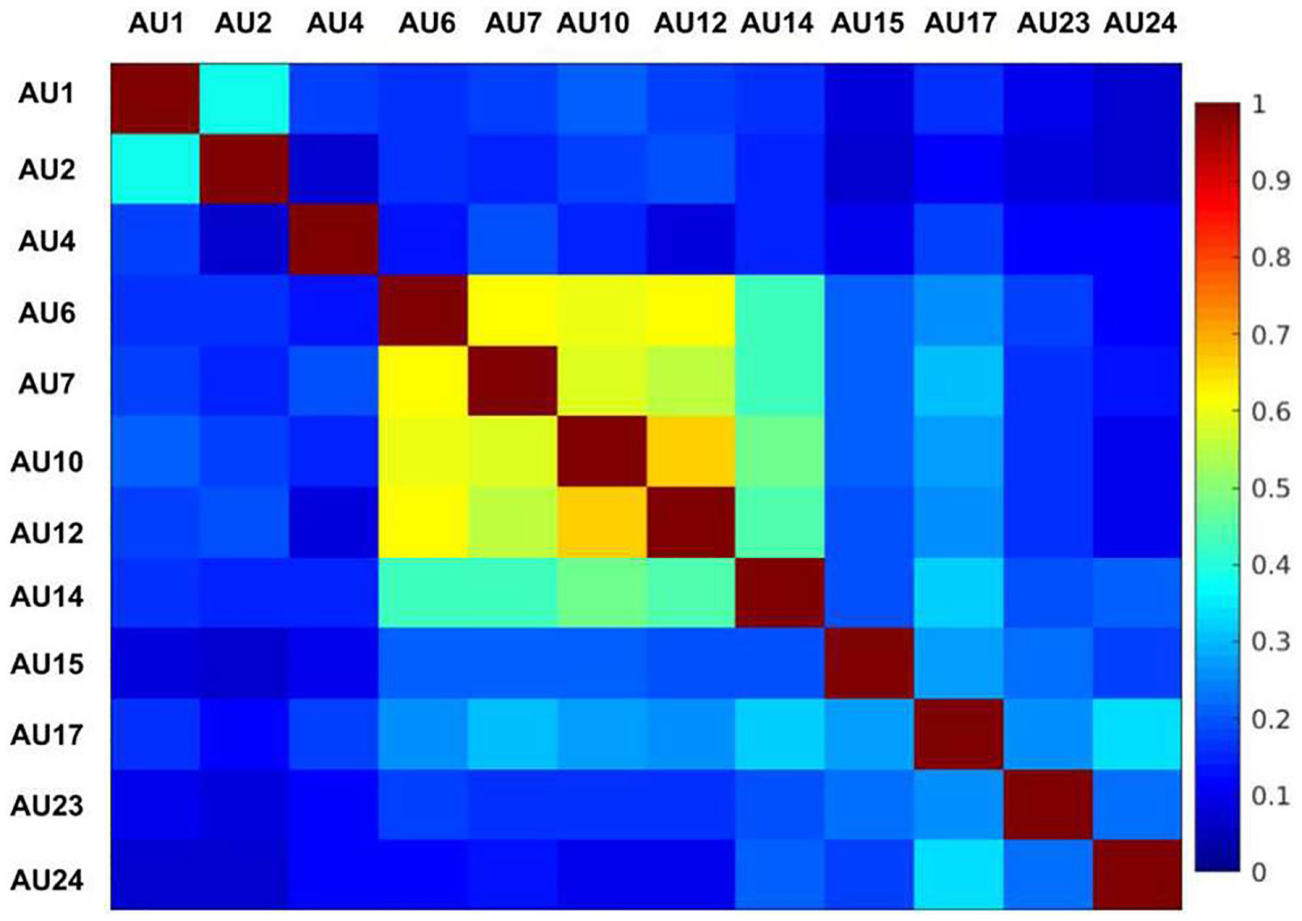
Co-occurrence matrix of AUs computed with Jaccard index.

BP4D is a manually FACS annotated database of spontaneous behavior containing 2D and 3D videos of 41 subjects (23 female and 18 male). Following previous research in AU detection, only 2D videos are used here. In BP4D, well-designed tasks initiated by an experimenter are used to elicit varied spontaneous emotions. Each subject performs eight tasks. In total there are 328 videos of approximately 20 s each that have been FACS annotated manually. This results in about 140,000 valid, manually FACS annotated frames. We include 12 AUs that occurred in more than 5% of the frames. Positive samples are defined as ones with intensities equal to or higher than A-level, and the remaining ones are negative samples. We visualize the co-occurrence matrix of AUs computed using Jaccard index in Figure 3. It can be observed that AU6, AU7, AU10, AU12, and AU14 co-occur frequently.

### 4.2. Network

In 2D-CNN, we employ 32, 64, and 64 filters of 5 × 5 pixels in three convolutional layers with a stride of 1. After convolution, rectified linear unit (ReLU) is applied to the output of the convolutional layers to add non-linearity to the model. We apply batch normalization to the outputs of all convolutional layers. The network contains three maxpooling layers that are applied after batch normalization. We apply max-pooling with a 2 × 2 window such that the output of max-pooling layer is downsampled with a factor of 2. At the output of the fully connected layer of static encoder, we obtain an encoding of size 1 × *M*, where *M* = 60.

In 3D-CNN, we select the patch sequence length *T* = 20. We employ 32, 64, and 64 filters of 5 × 5 × 5 pixels in the first two convolutional layers and 2 × 5 × 5 pixels in the final convolutional layer with a stride of 1. 3D convolutional layers are followed by ReLU and batch normalization layers. The first two batch normalization layers are followed by maxpooling layers with a 2 × 2 × 2 window, while the last batch normalization layer is followed by a maxpooling layer with a 1 × 2 × 2 window. At the output of the fully connected layer of dynamic encoder, we obtain an encoding of size 1 × *M*, where *M* = 60.

Temporal window length varies in the range [10, 24] in previous AU detection studies (Chu et al., 2017; Li et al., 2017). To be consistent with previous work, we selected patch sequences of length T = 20 within that range. The CNN architecture used in this study has been shown to be successful in previous studies (Cohn et al., 2018; Onal Ertugrul et al., 2019a,c). Two differences from previous work may be noted. One is the size of input images. Previously, we used holistic face images of size 200×200. Here we use local facial patches of size 100 × 100. The other difference results from the smaller input size. Because input size was reduced by 50%, we reduced the number of filters by 50% from 64, 128, and 128 filters to 32, 64, and 64 filters. The number of convolutional layers remained the same.

We obtain a patch encoding *e*_*p*_ of size 1 × 120, for each frame, which is obtained by concatenating 1×60 dimensional outputs of static and dynamic encoder outputs. In patch attention layer, we use the weight matrix *W*_*f*_ of size 120 × 120 and face level context vector *c*_*f*_ as 1 × 120. Attention layer output is a face encoding v of size 1 × 120, for each frame.

### 4.3. Training

We trained our architecture with mini-batches of 50 samples for 10 epochs. We used stochastic gradient descent (SGD) optimizer. Our models were initialized with learning rate of 1e-3, with a momentum of 0.9. In order to keep variability in the data, we used all of the available frames and did not subsample training frames to generate balanced dataset. For each AU, we assign *w*_*pos*_ to the ratio between the number of training frames excluding the AU and containing the AU. We perform a subject independent three-fold cross-validation for BP4D dataset. Our folds include the same subjects as in Zhao et al. (2016a).

### 4.4. Evaluation Measures

We evaluate network performance on two metrics: F1-score and area under the receiver operator characteristics curve (AUC). F1-score is the harmonic mean of precision (P) and recall (R) $\frac{2\text{RP}}{R+P}$. It is widely used in the literature and therefore enables comparison with the many approaches that have used it to report their performance. Because F1-score is highly attenuated by imbalanced data (Jeni et al., 2013), however, results for less frequent AUs must be considered with caution. AUC has the advantage of being robust to imbalanced data but has been reported less frequently in the literature. It supports more limited comparisons with other approaches.

### 4.5. Threshold Tuning

For each AU, our model predicts a value between 0 and 1, denoting the probability that the specified AU is present in the frame. In order to binarize the output, we take threshold τ = 0.5 and then evaluate the performance of D-PAttNet. Although during training we employed a weighted loss based on the baserates of AUs, it does not totally solve class imbalance problem. Optimal threshold τ may be different for different AUs and may not be equal to 0.5. We optimized the threshold τ ∈ [0.1, 0.9] on training set and evaluate the test performance in D-PAttNet^tt^.

## 5. RESULTS

### 5.1. Performance Comparison With the State-of-the-Art

We compare the performance of D-PAttNet with the following state-of-the-art approaches:

**Linear SVM (LSVM)** is based on training an SVM classifier using the SIFT features obtained from the frames without considering patch learning.
**Joint patch and multilabel learning (JPML)** (Zhao et al., 2016a) simultaneously selects a discriminative set of patches and learn multi-AU classifiers. It uses SIFT features obtained from patches.
**Deep region and multilabel learning (DRML)** (Zhao et al., 2016b) combines region learning and multilabel learning for AU detection.
**Network combining CNN and LSTM (LSTM)** (Chu et al., 2017) employs CNN to model spatial information and LSTM to model temporal dynamics in a sequential way for multilabel AU detection.
**Adversarial Training Framework (ATF)** (Zhang et al., 2018) is a CNN-based framework in which AU loss is minimized and identity loss is maximized to learn subject invariant feature representations during the adversarial training.
**Finetuned VGG Network (FVGG)** (Li et al., 2018) is the model obtained after finetuning the pretrained VGG 19-layer model.
**Network with enhancing layers (E-Net)** (Li et al., 2018) is the finetuned VGG network with enhancing layer which forces the network to pay more attention to AU interest regions on face images.
**Enhancing and Cropping Network (EAC Net)** (Li et al., 2018) is a pretrained CNN model with enhancing (E-Net) and cropping (C-Net) layers. E-net forces the network to attend more to AU interest regions based on a predefined attention map while C-Net crops facial regions around detected landmarks and applies upscaling and convolutional layers in the cropped regions.
**Deep Structured Inference Network (DSIN)** (Corneanu et al., 2018) is a deep network which performs patch learning to learn local representations and structure inference to model AU correlations.
**Joint AU detection and face alignment (JAA)** (Shao et al., 2018) is a deep learning based joint AU detection and face alignment framework in which multi-scale shared features for the two tasks are learned firstly, and high-level features of face alignment are extracted and fed into AU detection.
**Patch-attentive deep network (PAttNet)** (Onal Ertugrul et al., 2019b) is a CNN-based approach which jointly learns local patch representations and weights them with a learned attention mechanism for AU detection.

F1-score performances for the state-of-the-art approaches and D-PAttNet are given in Table 1. We also report results with Only3D-PAttNet, which includes only 3D CNN component of the D-PAttNet. Note that, for DSIN and D-PAttNet, superscript^*tt*^ denotes the results after tuning the threshold. For fair comparison, we excluded the studies which do not follow three-fold protocol (Tősér et al., 2016).

Results reflect that, D-PAttNet and D-PAttNet^*tt*^ give the best F1-score for 6 of 12 AUs (For D-PAttNet AU6, AU7, AU12, and AU23 and for D-PAttNet^*tt*^ AU15 and AU24). For the remaining 6 AUs (AU1, AU2, AU4, AU10, AU14, and AU17), D-PAttNet^*tt*^ gives the second best result. For four of the AUs (AU1, AU10, AU14, and AU17) for which D-PAttNet or D-PAttNet^*tt*^ did not perform the best, DSIN^*tt*^ show the best F1-score. On average, our method outperforms all of the comparison approaches and provides 2.1% absolute improvement over PAttNet.

Since F1-score is affected by the skew in the labels and some action units are highly skewed, we also compute AUC results, which are not affected by the skew. Only a few studies report AUC values. In Table 2, we compare the performance of D-PAttNet with the state of the art approaches using AUC. D-PAttNet gives an average AUC of 73.4% over all AUs. For each AU, AUC is above 64%. D-PAttNet gives superior performance compared to all of the approaches reporting AUC for 9 of the 12 AUs except for AU14, AU15, and AU24. For these three AUs, the maximum AUC is obtained for PAttNet.

Comparison of variants of PAttNet approach reflects that D-PAttNet which combines 2D CNN with 3D CNN outperforms PAttNet, which only has 2D CNN. Both variants give much better performance compared to using Only3D-PAttNet, which only has 3D CNN. D-PAttNet gives the best F1-scores for all AUs and the best AUC values for all but three AUs.

For the comparisons between D-PAttNet and other two variants (PAttNet and Only3D-PAttNet) we performed significance tests as given in Table 3. For each set of comparisons we controlled for Type I error using Bonferroni correction. With experiment-wise error of 0.05 and 12 comparisons in each set, a *p* of 0.004 is the critical value for significance. For AU7, AU10 and AU14 D-PAttNet significantly outperforms PAttNet when F1 scores are compared. When AUC values are compared, D-PAttNet performs significantly better for AU1, AU6, and AU7. Moreover, D-PAttNet outperforms Only3D-PAttNet for all AUs except for AU1 when F1 scores are compared. When AUC is used, it is significantly better for AU12, AU15, and AU24.

### 5.2. Performance Comparison of Using Sigmoid and Softmax Functions for Attention in Variants of Patch-Attentive Deep Networks

In this section, we compare the AU detection results of using our proposed attention function sigmoid and conventional activation function softmax to weight the contributions of patches. We compare these functions for (i) PAttNet approach which has 2D CNN to model static information, (ii) Only3D-PAttNet approach which has 3D CNN to model dynamic information, and (iii) D-PAttNet approach which combines static and dynamic information using 2D CNN and 3D CNN. We compare F1-scores and AUC values in Tables 4, 5, respectively. We also performed significance tests for the comparisons between sigmoid & softmax in given Table 6.

Comparison of the softmax and sigmoid rows of each approach in Table 4 shows that using softmax instead of sigmoid for both PAttNet and D-PAttNet causes a drop in the F1-scores for all AUs. Decreases in F1 are significant for all AUs except for AU24. For Only3D-PAttNet, sigmoid function performs similarly to softmax. We observe similar results for AUC values in Table 5. Decreases in AUC are significant for four AUs namely, AU4, AU12, AU15, and AU17. When we force the network to attend one or a few patches, it cannot learn proper facial representation. These results are consistent with the assumption that even if AUs relate to specific facial regions, co-occurring nature of AUs causes the contribution of other facial regions to detect specific AUs. When softmax attention function is used, D-PAttNet leads to a 2.4% increase in the average F1-score (see Table 4), and a1.7% increase in the AUC (see Table 5). Similarly, using patch dynamics provides a 1.5% improvement in the average F1-score (see Table 4) and a 0.7% improvement in the average AUC (see Table 5).

### 5.3. Patch Attention Analysis

**FIGURE 4 |:**
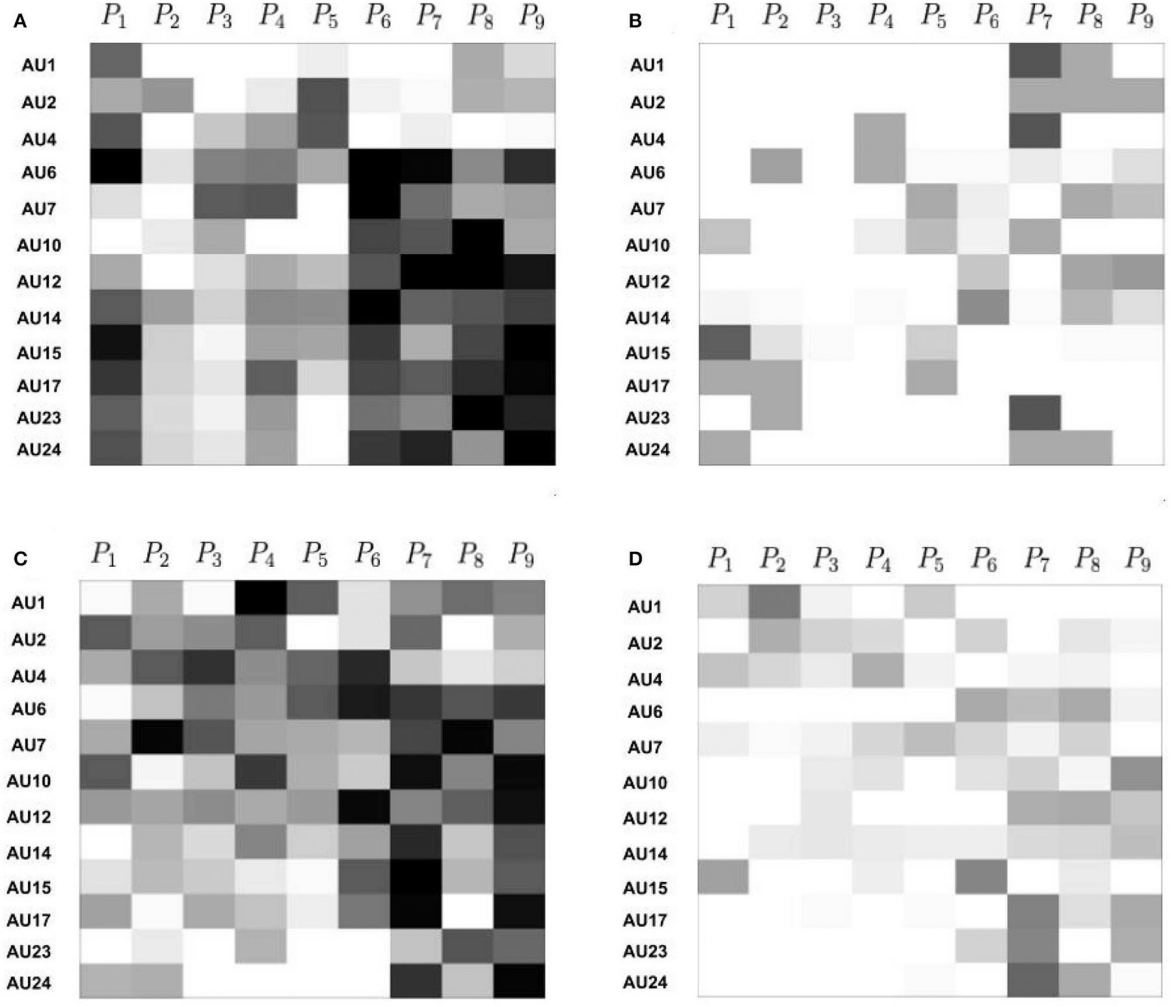
Average attention maps for PAttNet with sigmoid attention **(A)**, PAttNet with softmax attention **(B)**, D-PAttNet with sigmoid attention **(C)**, and D-PAttNet with softmax attention **(D)**. Attention maps are obtained by averaging attention weights of all samples. Attention weights are in [0,1]. White color represents no attention (0) and black color represents the maximum attention (1).

We visualize the attention maps formed using the learned attention weights of D-PAttNet with sigmoid attention, D-PAttNet with softmax attention, PAttNet with sigmoid attention, and PAttNet with softmax attention in Figure 4. We obtain an attention map for each sample and then average these maps to obtain the presented attention maps. In all maps, entries can take values between [0,1]. Cells with black color denote that the corresponding patch has high attention weight (is significant) to detect the corresponding AU for all of these folds whereas cells with white color denote that the related patch is not significant to detect the corresponding AU in any of the folds. Multiple patches contribute with varying weights to detect AUs.

#### 5.3.1. Comparison of Sigmoid and Softmax Attention

We can compare the attention maps obtained using sigmoid (Figures 4A,C) and softmax (Figures 4B,D) attention. As expected, we obtain denser maps with sigmoid attention for both PAttNet and D-PAttNet since softmax tends to select sparse entries. Moreover, we observe larger number of black or dark gray entries in the attention maps obtained using sigmoid meaning that models learned for different folds agree on the significance of corresponding patches to detect related AUs. On the other hand, attention maps obtained using softmax attention do not have black entries and have a few dark gray entries. This indicates an inconsistency between the models trained for different folds, each of which learns to detect the same AU from different parts of the face.

#### 5.3.2. Comparison of D-PAttNet and PAttNet

When we compare D-PAttNet with sigmoid (Figure 4A) and PAttNet with sigmoid (Figure 4C), we observe that for most of the AUs, the network learns to attend meaningful patches. In both maps, generally higher attention is observed in upper face patches to detect AUs of upper face region (AU1, AU2, and AU4). Similarly, higher attention is observed in mouth and lip corner patches to detect AUs of lower face region. In both maps, the highest attention is given to patches containing eyebrows (*P*_1_ for D-PAttNet and *P*_4_ for PAttNet) to detect AU1. AU12 is detected mainly from patches containing mouth and lip corner regions (*P*_7_, *P*_8_, and *P*_9_ for D-PAttNet and *P*_6_, *P*_9_ for PAttNet).

AU6 (contraction of the orbicularis oculi) raises the cheeks, narrows the eye aperture, and in social contexts, such as BP4D, typically occurs together with AU12 (zygomatic major). AU12 stretches the lip corners obliquely. Because AU6 and AU12 frequently co-occur and lip-corner stretching often is a relatively prominent appearance change, it may not be surprising that PAttNet for AU6 (Figure 4C) learns to attend more to patches containing lip corner, cheek, and mouth than to ones containing only the eyes. What is unexpected is that when patch dynamics are included for AU6 in PAttNet (Figure 4A), eye features become more salient (*P*_1_). The same effect may be seen with respect to AU7, which also is highly correlated with AU12 (*P*_6_ in Figure 4A and *P*_8_ in Figure 4C). The addition of dynamics in this way contributes to the detection of these AUs.

When we compare D-PAttNet with softmax (Figure 4B) and PAttNet with softmax (Figure 4D), we observe that forcing the classifier to attend sparse facial regions with softmax attention causes the network to attend irrelevant patches for some AUs in D-PAttNet. For example, to detect eye AUs, AU1 and AU2 the classifier does not attend to any of the eye patches. Recall that a black cell represents that the corresponding patch is significant to detect specific AUs for all or majority of the input frames. Neither maps for models with softmax attention contains black or dark cells. Contrary to the maps obtained with sigmoid atention, models with softmax attention do not attend to consistent patches to detect specific AUs for different images. Therefore, using softmax function for attention is not a good option for D-PAttNet and PAttNet.

## 6. DISCUSSION AND CONCLUSION

Inspired by the human perception of face and facial actions, we have proposed a dynamic patch-attentive deep network called D-PAttNet for AU detection. Analogous to OFA in human face perception, we encode local patches in an early stage of the network. Then, analogous to FFA, patch-based information is fused at a later stage by means of an attention mechanism. Analogous to STS, spatiotemporal dynamics are modeled by 3D-CNN.

In D-PAttNet, we first apply 3D face registration to remove the variation caused by the differences in pose and scale. Then, we crop patches containing important facial parts to detect specific AUs. We encode static patch information using 2D-CNN and patch dynamics using 3D-CNN and concatenate them to obtain patch encodings. After encoding each patch with CNN-based encoders, we weight the contribution of patch encodings using a patch attention mechanism. To allow multiple patches to contribute AU detection, we employ sigmoidal attention rather than the conventional softmax attention.

D-PAttNet outperforms state-of-the-art approaches on BP4D. Considering patch dynamics in D-PAttNet leads to an increase in the AU detection performance compared to its variants PAttNet and Only3D-PAttNet. Tuning the decision threshold of classifier further improves the detection performance. While D-PAttNet and PAttNet results are closer to each other, Only3D-PAttNet results are much worse than these two. Both PAttNet and D-PAttNet include a 2D CNN component. Current frame whose AUs are being detected is explicitly fed to these models through the 2D CNN component. However, in Only3D-PAttNet, 2D-CNN component does not exist. A sequence of frames is given as input to the 3D-CNN component but the task is to predict the AU occurrences of the last frame. Therefore, it may be more difficult for Only3D-PAttNet model to figure out the problem compared to the other variants.

Visualizing attention maps provides interpretation of the significant facial regions to detect AUs. Attention maps show that, with the help of sigmoidal attention D-PAttNet chooses to attend multiple patches and the most significant patches are meaningful. Softmax attention map is much sparser and leads to lower AU detection performance. While the facial regions attended in both D-PAttNet and PAttNet are similar, D-PAttNet is more successful to capture subtle appearance changes from the dynamics.

A limitation of our work is that we only tested our approach on a single database, BP4D, in which non-frontal variation in head pose is relatively limited. The 3D registration in D-PAttNet may be especially effective in databases that have larger non-frontal variation in head pose. More generally, generalizability of models and decision thresholds across databases or domains are open research questions. Decreases in classifier performance are common in cross-domain settings (Onal Ertugrul et al., 2019a) even when models are trained on large databases. Future work should explore cross-domain generalizability of models and thresholds in large databases that vary in pose characteristics. Another future direction would be modeling spatiotemporal patch dynamics for AU intensity estimation.

## Figures and Tables

**TABLE 1 |:** AU detection performances (F1-scores) on BP4D dataset.

AU	1	2	4	6	7	10	12	14	15	17	23	24	Avg.
LSVM	23.2	22.8	23.1	27.2	47.1	77.2	63.7	64.3	18.4	33.0	19.4	20.7	36.7
JPML	32.6	25.6	37.4	42.3	50.5	72.2	74.1	65.7	38.1	40.0	30.4	42.3	45.9
DRML	36.4	41.8	43.0	55.0	67.0	66.3	65.8	54.1	33.2	48.0	31.7	30.0	47.7
LSTM	31.4	31.1	**71.4**	63.3	77.1	45.0	82.6	72.9	34.0	53.9	38.6	37.0	53.2
ATF	39.2	35.2	45.9	71.6	71.9	79.0	83.7	65.5	33.8	60.0	37.3	41.8	55.4
FVGG	27.8	27.6	18.3	69.7	69.1	78.1	63.2	36.4	26.1	50.7	22.8	35.9	43.8
E-Net	37.6	32.1	44.2	75.6	74.5	80.8	85.1	56.8	31.6	55.6	21.9	29.1	52.1
EAC-Net	39.0	35.2	48.6	76.1	72.9	81.9	86.2	58.8	37.5	59.1	35.9	35.8	55.6
JAA	47.2	**44.0**	54.9	77.5	74.6	84.0	86.9	61.9	43.6	60.3	42.7	41.9	60.0
DSIN	**51.7**	40.4	56.0	76.1	73.5	79.9	85.4	62.7	37.3	62.9	38.8	41.6	58.9
DSIN^*tt*^	**51.7**	41.6	58.1	76.6	74.1	**85.5**	87.4	**72.6**	40.4	**66.5**	38.6	46.9	61.7
PAttNet	46.1	41.4	57.1	77.9	76.1	83.8	88.4	66.5	51.2	61.6	44.1	57.3	62.6
Only3D-PAttNet	36.8	33.9	47.9	74.6	72.2	81.7	84.0	62.0	41.9	58.1	40.0	45.7	56.6
D-PAttNet	50.4	41.1	58.4	78.6	77.5	84.6	89.0	66.7	**52.6**	64.5	49.0	**57.6**	64.1
D-PAttNet^*tt*^	50.7	42.5	59.0	**79.4**	**79.0**	85.0	**89.3**	67.6	51.6	65.3	**49.6**	57.5	**64.7**

The best results are shown in bold and the second best results are shown underlined. For methods DSIN and D-PAttNet, ^*tt*^ denotes the use of threshold tuning.

**TABLE 2 |:** AU detection performances (AUC) on BP4D dataset.

AU	1	2	4	6	7	10	12	14	15	17	23	24	Avg.
LSVM	20.7	17.7	22.9	20.3	44.8	73.4	55.3	46.8	18.3	36.4	19.2	11.7	32.3
JPML	40.7	42.1	46.2	40.0	50.0	75.2	60.5	53.6	50.1	42.5	51.9	53.2	50.5
DRML	55.7	54.5	58.8	56.6	61.0	53.6	60.8	57.0	56.2	50.0	53.9	53.9	56.0
PAttNet	66.5	65.6	74.4	78.6	71.8	78.4	86.4	**65.4**	**72.1**	70.1	68.0	**74.8**	72.7
Only3D-PAttNet	59.5	59.6	67.6	75.9	66.1	75.9	81.5	63.0	65.6	67.1	64.6	68.1	67.9
D-PAttNet	**68.3**	**66.0**	**75.6**	**79.1**	**73.0**	**79.0**	**87.0**	64.9	72.0	**71.9**	**69.5**	74.5	**73.4**

The best results are shown in bold.

**TABLE 3 |:** Significance of differences between D-PAttNet and the two other variants (PAttNet and Only3D-PAttNet) by *t*-test.

		1	2	4	6	7	10	12	14	15	17	23	24
D-PAttNet >	F1	n.s.	*	n.s.	n.s.	**	**	n.s.	**	*	*	n.s.	n.s.
PAttNet	AUC	**	n.s.	n.s.	**	**	n.s.	n.s.	**	n.s.	*	n.s.	n.s.
D-PAttNet >	F1	*	**	**	**	**	**	**	**	**	**	**	**
Only3D-PAttNet	AUC	*	n.s.	*	n.s.	n.s.	n.s.	**	n.s.	**	n.s.	n.s.	**

* *p* < *0.05*,

** *p* < *0.05/12*.

The latter are significant after correcting for multiple comparisons. n.s., not significant. Cells denoted with gray color indicates cases where the results for PAttNet are greater than the ones for D-PAttNet.

**TABLE 4 |:** Comparison of sigmoid and softmax attention functions in PAttNet, Only3D-PAttNet, and D-PAttNet (F1-scores).

AU		1	2	4	6	7	10	12	14	15	17	23	24	Avg
PAttNet (2D)	Softmax	37.2	28.4	41.4	73.4	69.8	79.3	81.9	58.7	32.7	58.5	39.8	49.2	54.2
	Sigmoid	46.1	**41.4**	57.1	77.9	76.1	83.8	88.4	66.5	51.2	61.6	44.1	57.3	62.6
Only3D	Softmax	46.5	33.8	41.3	74.5	71.4	81.9	85.9	57.6	33.4	55.2	43.1	46.1	55.9
PAttNet	Sigmoid	36.8	33.9	47.9	74.6	72.2	81.7	84.0	62.0	41.9	58.1	40.0	45.7	56.6
D-PAttNet (2D + 3D)	Softmax	42.5	41.2	42.0	72.1	72.2	82.6	86.7	62.1	32.2	54.9	37.8	52.4	56.6
	Sigmoid	**50.4**	41.1	**58.4**	**78.6**	**77.5**	**84.6**	**89.0**	**66.7**	**52.6**	**64.5**	**49.0**	**57.6**	**64.1**

The best results are shown in bold.

**TABLE 5 |:** Comparison of sigmoid and softmax attention functions in PAttNet, Only3D-PAttNet, and D-PAttNet (AUC).

AU		1	2	4	6	7	10	12	14	15	17	23	24	Avg
PAttNet (2D)	Softmax	59.2	54.7	63.4	74.7	65.9	72.9	76.9	58.8	59.6	67.3	65.3	71.4	65.8
	Sigmoid	66.5	65.6	74.4	78.6	71.8	78.4	86.4	**65.4**	**72.1**	70.1	68.0	**74.8**	72.7
Only3D	Softmax	66.4	60.2	62.4	74.8	65.1	78.8	83.6	59.5	59.3	64.5	67.1	69.4	67.6
PAttNet	Sigmoid	59.5	59.6	67.6	76.0	66.1	75.9	81.5	63.0	65.6	67.1	64.6	68.1	67.9
D-PAttNet (2D + 3D)	Softmax	63.2	63.9	60.7	73.2	63.5	77.0	84.4	61.4	59.1	63.2	63.4	77.4	67.5
	Sigmoid	**68.3**	**66.0**	**75.6**	**79.1**	**73.0**	**79.0**	**87.0**	64.9	72.0	**71.9**	**69.5**	74.5	**73.4**

The best results are shown in bold.

**TABLE 6 |:** Significance of differences between classifiers (sigmoid and softmax) by *t*-test.

		1	2	4	6	7	10	12	14	15	17	23	24
Sigmoid >	F1	**	**	**	**	**	**	**	**	**	**	**	n.s.
Softmax	AUC	n.s.	n.s.	**	n.s.	n.s.	n.s.	**	n.s.	**	**	*	*

* *p* < *0.05*,

** *p* < *0.05/12*.

The latter are significant after correcting for multiple comparisons. n.s., not significant. Cells denoted with gray color indicates cases where the results for softmax are greater than the ones for sigmoid.
